# Spontaneous Intracranial Hypotension Manifesting Orthostatic Headache Worsened by Playing the Saxophone and Treated by Japanese Herbal Kampo Medicine Goreisan

**DOI:** 10.7759/cureus.25393

**Published:** 2022-05-27

**Authors:** Masahito Katsuki, Kenta Kashiwagi, Shin Kawamura, Akihito Koh

**Affiliations:** 1 Department of Neurosurgery, Itoigawa General Hospital, Itoigawa, JPN; 2 Composer and Singer, Song Writer, Cuty KATSKI Music Office, Niigata, JPN; 3 Department of Neurology, Itoigawa General Hospital, Itoigawa, JPN

**Keywords:** suidoku, magnetic resonance imaging, cerebrospinal fluid (csf), aquaporin-4, glymphatic system, wind instrument, orthostatic headache, kampo medicine (japanese herbal medicine), spontaneous intracranial hypotension (sih), goreisan (tj-17)

## Abstract

We present a 15-year-old Japanese girl with no previous medical history who presented with a gradually worsening series of orthostatic headaches. We diagnosed spontaneous intracranial hypotension, worsened by playing the saxophone and its Valsalva maneuver effect. She was treated with Japanese herbal *Kampo *medicine *Goreisan* 7.5 g/day in three divided doses, and her symptoms gradually improved. Her headache has never recurred for a year when she played the saxophone. Our case’s headache may have been further exacerbated by cerebrospinal fluid (CSF) leakage due to CSF pressure increase by Valsalva maneuvers while playing the saxophone. Our case suggested that the Japanese herbal *Kampo* medicine *Goreisan* can facilitate the glymphatic system and adjust the CSF pressure appropriately.

## Introduction

Intracranial hypotension typically manifests as an orthostatic headache with neck pain, dizziness, and fatigue. The posture has an impact on its orthostatic headache. The headache continues daily, and for which the medication is less effective. Cerebrospinal fluid (CSF) leakage is the most common underlying factor. The idiopathic or traumatic rip of the dura matter and subsequent leakage is thought to be caused by structural weakness in the dura matter [1]. According to The Japanese Society of Cerebrospinal Fluid Leak Official Guideline [1], traumatic intracranial hypotension is defined to have an onset of 30 days after the trauma. While spontaneous intracranial hypotension occurs more commonly in women in their late 30s, and its annual incidence is estimated to be five per 100,000. The leakage site can occasionally be identified by magnetic resonance imaging (MRI). Rest, hydration, or epidural blood patch (EBP) with blood or fibrin sealant are the most common treatments, while surgery is sometimes required. The majority of patients had favorable outcomes. However, delays in diagnosis are caused by a variety of presenting signs and symptoms, as well as a lack of understanding of the illness [2].

We herein describe a 15-year-old girl who presented an orthostatic headache due to spontaneous intracranial hypotension, which is aggravated by playing the wind instrument, the saxophone. Besides, we could treat the orthostatic headache with the Japanese herbal *Kampo* medicine *Goreisan *without rest, admission, or surgery. This is one of the rare cases of headache worsened by the Valsalva maneuver due to playing the wind musical instruments [2-4] and which could be treated by *Kampo* medicine, *Goreisan* [5,6].

## Case presentation

A 15-year-old Japanese girl with no previous medical history appeared with a year’s worth of increasingly severe orthostatic headaches. There was no additional trauma in her past. She started playing the saxophone at the age of 13 years and practiced it in the school band every day. The orthostatic headache first appeared only when she practiced saxophone with Valsalva maneuver. From the age of 14 years, it began to present for a while after she stood up without playing the saxophone. It gradually aggravated from the age of 15 years, and daily activity was disturbed. Her orthostatic headache was bilateral, with a numerical rating scale (NRS) of 8/10 and no pulsing symptoms. Every day, she had a headache. No nausea, photophobia, or phonophobia was present. The headache did not develop while lying down or sitting, but only after standing for a few minutes. She claimed that her headache got worse as she played the saxophone while standing and sitting. Coughing and toileting did not make the headaches worse.

**Figure 1:**
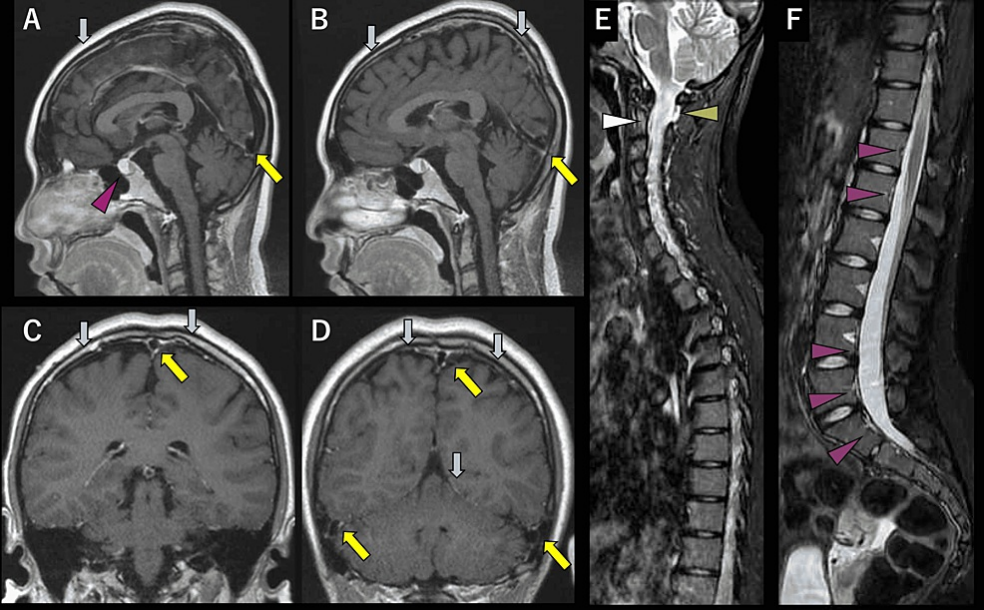
Magnetic resonance imaging

The gadolinium contrast-enhanced T1-weighted MR imaging showed partially enhanced dura matter (blue arrows in Figure 1A-1D), venous distension sign (yellow arrows in Figure 1A-1D), and pituitary enlargement (red arrowhead in Figure 1A). Enlarged intracavernous sinus, subdural fluid correction, low-lying tonsils, effacement of the suprasellar cistern of 4.0 mm or less, effacement of the prepontine cistern of 5.0 mm or less, or mamillopontine distance of 6.5 mm or less was not found. The spinal short inversion-time inversion recovery images showed the C1/2 sign (green arrowhead in Figure 1E) and epidural venous plexus (white arrowhead in Figure 1E). The incomplete floating dural sac sign was shown (purple arrowhead in Figure 1F). The dinosaur tail sign was not found [1,7]. We did not perform a more accurate modality for leakage localization, such as computed tomography-myelography, considering the radiation exposure and her young age.

We diagnosed spontaneous intracranial hypotension from the medical history and the MRI findings, which was worsened by playing the saxophone. She wanted to continue playing saxophone and did not want to be admitted. Therefore, we prescribed 7.5 g of Japanese herbal *Kampo *medicine *Goreisan* to treat *suidoku* status (fluid disturbance such as edema, dehydration, and dislocation) [5,6,8] in three divided doses. The headache severity slightly improved over one month. After one month, her headache was relieved as her NRS score was 2/10, and it occurred two times per month from the next month. Then, her headache has never recurred for one year, and she can still now play the saxophone, intaking *Goreisan* 7.5 g/day in three divided doses. Her family doctor will reduce *Goreisan* to 5.0 g/day in two divided doses, 2.5 g/day as a single dose, and 2.5 g as needed over some years. The follow-up MRI will be performed in the future.

## Discussion

Intracranial hypotension and playing wind instruments

This is one of the uncommon cases of headache caused by intracranial hypotension exacerbated by the Valsalva maneuver while playing saxophone. The saxophone requires an expiratory pressure of up to 80 cmH_2_O [9]. Although the value is not particularly high, playing a musical instrument necessitates a high level of expiratory pressure ​[2]. Valsalva maneuver induced by these expiratory efforts seems to have a role in headache exacerbation. Previously, four cases have been reported whose headaches were worsened by playing wind instruments (Table 1) [2-4]. The common mechanism for playing wind instruments, which worsens the headache, is Valsalva maneuvers that interfere with venous drainage. The disturbed venous drainage increases the intracranial pressure, leading to more CSF leakage or headaches.

**Table 1:** Previous reports on intracranial hypotension and playing wind musical instruments

Author	Year	Age	Sex	Instrument	Comorbidity	Symptom	Treatment
Patrick et al. [2]	2007	40	Woman	Bagpipe	Chiari malformation I	Postural headache and neck stiffness	Lumbar 20 mL blood patch
Martínez-Lage et al. [3]	2013	10	Man	Cornet	Chiari malformation I	Growth retardation, headache aggravated with cough and playing cornet	Decrease his musical activity
Martínez-Lage et al. [3]	2013	10	Man	Horn	Hydrocephalus due to a block at the 4th ventricle outlets	Headache and vomiting for 2 days aggravated with playing horn	Endoscopic 3rd ventriculostomy
Katsuki et al. [4]	2022	13	Woman	Trombone	-	Orthostatic headache	Bedrest and hydration for 14 days
Ours	2022	15	Woman	Saxophone	-	Orthostatic headache	*Goreisan *7.5 g/day

The headache in our situation could have been made worse by CSF leakage caused by increased CSF pressure from Valsalva maneuvers while playing the saxophone. We believe that musicians who play wind instruments that need a high expiratory pressure are at risk of intracranial hypotension aggravation. When such music players see their doctors about postural headaches, the potential of spontaneous intracranial hypotension should be explored.

*Goreisan* for intracranial hypotension

We treated intracranial hypotension with *Goreisan *7.5 g/day, without rest, admission, or surgery. Our case suggested that *Goreisan *can be an alternative internal medicine for intracranial hypotension.

Takahashi and Mima first reported the efficacy of *Goreisan* for intracranial hypotension. They reported 18 patients diagnosed with CSF hypovolemia and prescribed* Goreisan* after EBP. Of the 18 patients, seven (39%) showed good recovery, and five (28%) showed moderate recovery over six months. They suggested that *Goreisan* after EBP may be effective in treating CSF hypovolemia [5].

Sayama et al. then reported the efficacy of some Japanese herbal *Kampo *medicines for intracranial hypotension after EBP. They investigated 19 patients who were treated with *Kampo *medicine, and 17 of them underwent EBP before. All the patients' symptoms improved, and nine had *Goreisan*, six *Kakkonto*, three *Keishikajutsubuto*, and two *hochuekkito*. They suggested the efficacy of *Goreisan* for intracranial hypotension patients [6].

Katsuki et al. reported a case with intracranial hypotension first treated by *Goreisan*, but the headache did not relieve. Therefore, the patient was admitted with rest and hydration, and the headache improved. They suggested that intaking *Goreisan, *combined with rest and hydration, can cure the headache [4].

We hypothesized the mechanism through which *Goreisan* works to treat intracranial hypotension. From a Japanese *Kampo* perspective, intracranial hypotension is hypothesized by some* Kampo *physicians to be linked to *suidoku* (fluid disturbances such as edema and dehydration). This is because* shin-eki *(all the fluid in the body) includes CSF, and *suidoku *means the *shin-eki'*s excess, reduction, and dislocation [5,6]. *Goreisan* is used to relieve headaches caused by *suidoku*, and it may work by inhibiting the water channel aquaporin-4 (AQP4) [8]. The glymphatic system in the brain is a fluid clearing mechanism. The perivascular spaces (PVS) of the major leptomeningeal arteries are where CSF from the subarachnoid space enters the brain. CSF is forced into the brain parenchyma through the Virchow-Robins spaces as the vascular tree branches. CSF then passes across the brain parenchyma’s glial basement membrane and astrocyte endfeet. AQP4 controls the glymphatic system. Endfeet of astrocytes express AQP4, which helps CSF flow into the brain parenchyma, where it mixes with the interstitial fluid. A polarized net fluid movement directed towards the venous PVS and perineuronal regions disperses the fluid in the interstitium. Finally, CSF emerges from the cranial and spinal neurons’ perineural sheaths, meningeal lymphatic veins, and arachnoid granulations. If the swollen astrocyte endfeet shut the PVS, CSF circulation is disrupted [10].* Kampo* medication* Goreisan *is thought to increase glymphatic flow by inhibiting AQP4 and thereby enabling CSF circulation by shrinking astrocyte endfeet and resolving PVS closure [8]. Although it is only a guess, increased CSF circulation could have rectified the excess CSF that had seeped through the dura. CSF circulation, the glymphatic system, and intracranial hypotension all require more research.

We discuss other possibilities why *Goreisan* had efficacy. *Goreisan* is composed of five herbal components. It is used to adjust the body's water balance by inhibiting mainly AQP4 channels and other AQP subtypes [11]. AQP3, 4, and 5 upregulates chemokine production [12], and *Goreisan* has a potent anti-inflammatory effect inhibiting these AQPs [13]. This anti-inflammatory may have somehow improved orthostatic pain. Besides, animal experiments using mice have shown that* Goreisan* has diuretic action when the body is overhydrated, and anti-diuretic action when dehydrated. Therefore,* Goreisan* is involved in regulating water metabolism and the maintenance of homeostasis [14]. It is possible that the internal administration of* Goreisan *has an antidiuretic effect and improves dehydration, resulting in spontaneous remission of the disease.

## Conclusions

We experienced the case with an orthostatic headache due to intracranial hypotension exacerbated by the Valsalva maneuver while playing saxophone. She did not have any history of major trauma, so we diagnosed spontaneous intracranial hypotension. When we see musicians who play wind musical instruments with orthostatic headaches affected by their postures, the possibility of spontaneous intracranial hypotension should be considered. Japanese herbal *Kampo *medicine *Goreisan* may be an alternative therapy for intracranial hypotension without rest, hydration, or surgery.
